# Psychiatry, Sex, and Science: The Making of “Adolescent” Motherhood in Southern Brazil

**DOI:** 10.1080/01459740.2017.1313252

**Published:** 2017-05-04

**Authors:** Dominique P. Béhague

**Affiliations:** ^a^ Medicine, Health and Society, Vanderbilt University, Nashville, Tennessee, USA; Department of Global Health and Social Medicine, King’s College London, London, UK; and The London School of Hygiene and Tropical Medicine, London, UK

**Keywords:** Adolescence, class, local biology, ontological politics, psychiatry, teen pregnancy

## Abstract

Research linking teen motherhood to psychoneurodevelopmental causes and pathologies has proliferated in the past two decades. In Brazil, a psychodevelopmental project of teen motherhood has gained traction despite many experts’ long-standing commitment to psychodynamic psychiatry and social epidemiology, generating epistemic tension rather than substitution. Drawing on historical ethnography conducted in Southern Brazil, I explore how this project materialized through the co-production of epistemic struggles, remedial interventions, and ontological politics. In showing how this co-production became interwoven with incremental changes in young women’s emotions, sexualities, relationships, and bodies, I describe how one particular “kind” of teen motherhood emerged and became entangled with both psychiatric knowledge-production and the angst of working-class political agency. In giving women a contested psychiatric language with which to rework their social–moral worlds, I argue that science did more than conceptualize teen childbearing in pathological terms; it contributed to its troubled transformation.

For over three decades, a promising experiment in community-based social psychiatry has been taking shape in Brazil. This experiment gained speed after the end of Brazil’s twenty-year dictatorship (1984) with the constitutional revision of 1988 which initiated the psychiatric de-institutionalization movement of the 1990s and 2000s. Rather than marginalize psychoanalysis in favor of cognitive behavioral psychology or neurobiology, as has largely been the case in the United States, Brazilian psychiatrists have embraced clinical and epistemic hybridity. Rekindling long-standing commitments to psychoanalytic psychiatry and Marxist-inspired social medicine, they have forged partnerships with leaders of civil society, many of whom worked to legitimize the political voice of the working-class during the dictatorship (Tenorio 2002). With a universal state-funded health care system that provides mental health care services through a decentralized network of clinics and schools, many placed near or in shantytowns, rates of uptake across all age-groups increased quickly, particularly in the southern more prosperous regions where this study took place. In a 2001 survey of 19-year olds, for example, upwards of 30 percent of those surveyed had seen a therapist in their lifetime, a rate that decreased to only 25 percent for those of very low socioeconomic income (Béhague 2009).

Since the mid 1990s, I have been studying how community psychiatry has unfolded and influenced the lives of young people in Pelotas, a small city in Southern Brazil well known for its comparatively high concentration of psychiatrists, psychologists, and leading physician/social epidemiologists. In the 1980s and 1990s, young people represented both a problematic and inspirational patient population for many Pelotense experts. This was partly because psychiatric approaches to young people’s mental health had become progressively more biomedical in countries powerful enough to influence global trends, such as the US and UK. In watching this biomedical episteme gain global traction with the renaissance of clinical–developmental notions of “adolescence” and the establishment of child- and adolescent-specific diagnoses such as attention deficit disorder and conduct problems, Pelotense psychiatrists became increasingly protective of the “social” in their work. Many watched with great concern as adolescent sexual life began acquiring a psychopathological dimension in the Anglophone literature, and as adolescent pregnancy in particular came to be understood as both resulting from neurodevelopmental risk taking and impulsivity, and a risk factor for comorbidities in adolescence and adulthood such as drug addiction, violence, conduct disorder, reproductive ill-health, and lasting major depression (Schmidt et al. 2006). Most Pelotense experts I knew considered adolescent pregnancy and motherhood, so clearly linked to poverty and low education, to be among the most insidious objects of pathologization. Young people, they explained, were promising and vibrant users of social psychiatry and they needed protection from such reductionist forms of medicalization. Moreover, many young patients began experiencing transformative therapies that spoke not of biology or disease, but of life histories, poverty, and political justice (Béhague 2009).

That was the 1990s. By the early 2000s, however, I began noticing that young women were more likely to be referred to their school’s psychologist by teachers if they were deemed too “demonstrative,” particularly in relation to sexuality. Around the same time, a number of small scale initiatives in schools began identifying sexual risk taking, inattention, and conduct problems as targets for the *prevention* of reproductive and mental health outcomes. In the mid 2000s, I began meeting teen mothers who understood their struggles not in relation to poverty, singlehood or marital strain, but as “depression” and even “post-partum depression”; some linked this to prior adolescent developmental problems. From the late 2000s, publications in Pelotas and elsewhere in Brazil began reporting on the lasting psychological effects of teen childbearing using adolescence-specific diagnostic tools taken directly from the biopsychiatric canon in the US and the UK (Chalem et al. 2012). Over these two decades, the professional landscape evolved in ways that were antithetical to all that social psychiatrists had worked towards.

How did adolescent sexuality, pregnancy, and motherhood become accepted loci of psychiatric expertise despite strong initial resistance? One common answer points to the globalization of Anglophone biomedical psychiatry, and the question of how populations are locally governed. The pathologization of teen pregnancy has traditionally been more intense in countries such as the US, UK, and South Africa, where public discourse surrounding teen pregnancy divert policy attention from the social and economic policies needed to ensure equity (Koffman 2012). In Brazil certainly, the reproductive and mental life of the poor have long been institutionalized concerns of the medical elite, and in recent decades, alarmist public discourses surrounding the sexuality of low-income teens have intensified (Carrara and Russo 2000).

Yet, I had a niggling sense that this framing was too top heavy for the reality I was observing, too centered on the rise of a subjugating biopsychiatric episteme. In this article, I explore the myriad of forces that led up to the biopsychiatric articulation of adolescent pregnancy in Pelotas. I draw from long-term fieldwork that I began in the late 1990s with first, an array of over 100 experts, several of whom I have interviewed repeatedly; and second a longitudinal study of 96 young people (and their parents, siblings and friends) selected from the 1982 Pelotas birth cohort study, whom I and a team of assistants visited repeatedly from ages fifteen (1997) to twenty-five (2007).^1^ Of these 96, 45 were girls, and of these 10 became teen mothers at some point between 1996 (14 years of age) and 2001 (19 years of age). These young women introduced me to another 15 teen mothers whom I also came to know over a long period of time.^2^

As I watched these women’s lives unfold, I learned that science and the clinic were not driving engines in what eventually became a psychologized science of adolescent pregnancy and motherhood. Rather, I came to conceptualize science and the clinic as midway points, recurring stopping points even, in much larger journeys. My analytical approach takes inspiration from the works of scholars who seek to move away from teleological analyses of science centered on how the power-knowledge nexus constructs its objects (Rajan and Leonelli 2013). Scientific objects are, rather, *co-constituted* in multi-directional relationships between clinic, science, and the everyday (Jasanoff 2012). As Bruno Latour has argued, some expert projects become “real” not by “grand design” but through the accumulation of “little solidities, little durabilities, little resistances” (Latour 1996:45). “A technological project,” he writes, is not transposed into “a context—it gives itself a context” (1996:133).

In finding my way through the forest that is this context-making story, Ian Hacking’s *Mad Travelers* (1998) has been particularly helpful. In this book, Hacking charts the convergence of synergistic “vectors” responsible for creating the particular “niche” that led to the short-lived appearance in the 1890s of a “fugue state” among travelling men (Hacking 1998). “Fugue” became a diagnosable entity, Hacking argues, because it invited controversy in the existing medical taxonomy, caught the public imaginary by fitting within a core cultural polarity of the times, was observable, and provided a certain class of people with release and escape. Hacking uses the mathematical metaphors “niche” and “vector” to underscore their synergistic and not merely summative nature; it is the specificity of this synergy that accounted for fugue’s contingent time-place appearance.

Below, I describe the context-producing “niche” that transformed adolescent pregnancy into a diagnosable psychiatric problem in Pelotas. In tackling the question “What is adolescence?” I investigate the birth of “adolescence” as a clinical category that enabled both “diagnosability” and taxonomic polemics. I then explore the epistemic struggles that the pathologization of adolescence conjured for experts, clinicians, teachers, families, and young people alike. I demonstrate how constructivist and essentializing logics surrounding the epistemic validity of adolescence as a clinical–developmental category facilitated numerous controversies, relating not just to science and expertise, but also to a pervasive “tradition versus modern” polarity that many low-income families grappled with and articulated in terms of the “strains of modern life.” “Adolescence,” simply put, became a “good to think with” an object of *bricolage*, to use Lévi-Strauss’s (1966) expedient metaphor (cf. Harding 1996).

Not all young women were equally interested in engaging with this good to think with object. In the section on “ontological politics,” I focus on a specific group of young women for whom debates on the epistemic validity of adolescent development became highly meaningful. These women’s epistemic interrogations, a counter point to the normativity of adolescence, also became conduits for experimenting with new ways of being and becoming, for realizing their desires to be “ethically otherwise” (Povinelli 2011), for finding “release” from the injustices of, as they put it, “modern life.” Such new ways of becoming took an emotional toll. Here I seek to expand on Hacking’s niche metaphor to call for a more explicit consideration of the role of new ontologies and ways of becoming as a key vector in making a scientific-clinical project “stick.”

I take seriously the possibility that teen pregnancy became inextricably linked to lasting psychological suffering for *some* women,^3^ not inherently so, in the way biopsychiatric experts might claim, but materially and vitally real nonetheless (Rose 2013). As I have shown elsewhere, the emotional turmoil that some mothers experienced was shaped not first and foremost by poverty, or even single motherhood, as critics of medicalization often argue (Béhague et al. 2012). Rather, emotional turmoil was concentrated amongst young mothers who were critical of the world around them, who put themselves in the line of fire of the classism they experienced, and who eventually embraced teen motherhood as a prideful anxious-ridden act of working-class defiance. This convergence of vectors, I will show, created a “situated biology” (Lock 2015)—a new “kind” of teen motherhood altogether (Hacking 1995)—that, in turn, contributed to the making of a new clinical-scientific project.

## What is “adolescence”?

Historians often trace the origins of a clinical notion of adolescence to the rise of evolutionary theory in the second half of the nineteenth century in Western Europe. In drawing conceptual links between the psyches of children and various classes of “sub-human primitives,” including poor people, Africans, and women, evolutionary theorists viewed child development as “recapitulating” the historical record of human-societal evolution more generally (Bowler 1989). Since the developmental phases of individuals in more “civilized” societies were said to be more complex and compressed than those in “primitive” societies, problems such as “feeblemindedness” (cognitive deficiencies) in children and adults were defined as a form of developmental regression toward primitivism. By the early twentieth century, intelligence and multidimensional measurement tools were devised to distinguish between normal and abnormal development, and through this, scientists began linking mild developmental delays in childhood to future adult pathology. Assessments of sexual development gained prominence, feeding into well-known eugenic proposals for the forced sterilization of those deemed psychologically and cognitively “unfit” for motherhood (Luker 1996).

Not all evolutionary theorists were in agreement with the strict Mendelian principles that underpinned eugenic proposals. Long-standing nature–nurture debates were repeatedly rekindled, particularly by scientists allied with late-nineteenth-century progressive movements who were critical of biological determinism (Neubauer 1992). “Adolescence” arose in this epistemic milieu. A central figure was German–American psychoanalytic psychologist Stanley Hall (1905), who extended the duration of childhood by defining adolescence as a period of necessary “metamorphic” psychological “storm and stress” that arose from the conflicted experience of being pulled towards both childish “primitivism” and adult “rationality.” Though deeply influenced by evolutionary theory, Hall was a Lamarckian: he rejected biological determinism and argued for the possibility of “evolutionary” improvement in individuals—and societies—through environmental “sanitation.” For him, adolescence represented a prime opportunity to rectify “bad biology” through reforms such as universal education, welfare, and psychological intervention. According to some historians, this more “optimistic” view of human development harked back to Romantic and anti-rationalist ideas that pre-dated evolutionary theory and were taken up with greater fervor in the Americas, including Brazil, than in Europe (Stepan 1991). Supporting these tendencies were critical strands of psychoanalytic theory that prevailed in the Southern cone, including Southern Brazil and Argentina, throughout the twentieth century (Tenorio 2002).

Clinical views of adolescence did not of course arise in a vacuum. Modern notions of child development were integral to the rise of industrial capitalism in the mid to late nineteenth century in Western Europe and North America (Ariès 1962; Comaroff and Comaroff 2006). During this time, large-scale structural changes—the passing of compulsory education and child-labor laws, and the population-wide institutionalization of schooling—prolonged the period between childhood and adulthood and dramatically changed the contexts in which children matured, from family life and apprenticeships to formal education, organized and mandated by the state. Though rural families and the urban poor often vociferously resisted the legal–institutional transformation of family life, the professionalization of child-rearing became increasingly normative with urbanization, rising education levels, women’s emancipation movements, demographic shifts towards reduced family sizes, and not least, the rise of a medical-psychiatric apparatus focused on redressing “developmental delays.” The institutionalization of child development was of course not universal and well-to-do children were—and continue to be—the most likely to experience the protracted childhoods and adolescences described in textbooks (Lesko 2001). Nevertheless, adolescence became an ontologically distinct experience shaped by the co-convergence of structural changes and a newly emerging language of development.

This history is important to sketch because it was foundational in setting normative standards for what it means to “develop” and “modernize”– and because it continues playing itself out in vastly different permutations across the globe. In Pelotas, clinical and pedagogic framings of adolescence are by no means new. In making sense of adolescent development, therapists and researchers consistently reference a diverse range of theorists such as Sigmund and Anna Freud, Melanie Klein, Erik Erikson, Paulo Freire, Eric Fromm and Emile Durkheim. Yet as noted, most experts have remained cautiously skeptical about “medicalizing” this phase of life. In fact, I would argue that the professionalization of adolescent development began not with expertise per se, but with structural-institutional changes taking place since the 1990s in Southern Brazil. All-age fertility rates have declined sharply, making families smaller, and upward mobility, though by no means common, has been experienced by an unprecedented proportion of the Brazilian population (Victora et al. 2011). Primary school enrollment rates have increased rapidly and are now near universal; various initiatives have improved access to education for girls. Continual waves of rural–urban migration into rapidly growing shanties have accentuated a now recognizable generation gap. This same period has been infused by the rise of a global youth culture transmitted in the media, within which clinical notions of adolescent “storm and stress” abound. And universal access to mental health services, with a large number of schools housing a psychologist or psychopedagogic specialist and with primary care clinics employing psychiatrists, has provided a ready-made platform for families and therapists to begin talking about and grappling with “adolescence.”

Not surprisingly, the vast majority of working-class parents I met spoke of adolescence as a “new thing”—a thing of *a vida moderna* (modern life). Many considered adolescence to be a stepping stone for achieving upward mobility and when their children faltered in school or struggled emotionally, as many invariably did, parents often sought psychological and psychiatric help. Even so, the search for upward mobility via “adolescence” was also rife with conflict and ambiguity. Parents told me that they felt pressured by educators to provide their children with a “protected” and unduly prolonged childhood by “making school the child’s only job,” delaying their participation in household chores and part-time work until well into their teen years. This often added strain to the way households were organized and could compound economic hardship. Parents routinely provided me with examples of families who had invested heavily in their children’s development with only debt to show. For young men, future heads of households, extended schooling and delayed employment were considered risky, a form of deskilling that impeded informal apprenticeships and the creation of personal networks needed to maintain an income in the informal sector. Over time, parents also became skeptical of school-based psychological services, which they argued singled out low-income students and typically countered their own parenting practices.

The social dilemmas linked to “adolescence” were particularly acute for parents of young women, not least because the growing focus on girls’ education challenges gender and kinship norms that many families hold dear. When parents described the benefits of education, for example, several focused on how schooling safeguards a girl’s moral/sexual reputation and marriage potential by separating “good” girls (*certinhas*) who stay in school and have few boyfriends, from *froxas*, “loose” girls who drop out of school and are sexually active. At the same time, parents worried that an overly studious and prolonged adolescence might keep their children away from the social environments that teach them about courtship right when the numbers of eligible young men begins to decline, thereby impeding their daughters’ marriage potential and leaving them childless. In fact, though all young women felt pressured to delay marriage in favor of education, adolescent pregnancy was not the life-shattering experience that public health discourses often assume it to be since it has the potential to become a core part of a successful courtship process (Heilborn et al. 2007).^4^ Indeed, the majority of teen mothers become pregnant before marriage but with their future partners (Gonçalves and Gigante 2006).

Even so, many families of low-income avidly sought education for their daughters, hoping for university entry even, despite the social risks this entailed, and many mothers adopted an approach to child-rearing that, as they explained, departed from the more *conformada* (conformist), *acomodada* (passive) and “traditional” ways in which they had been reared. These mothers routinely spoke to their daughters about the importance of staying in school and living out their “adolescences” to their fullest potential. Motivating this “nonconformism” was the search not just for economic stability but, more importantly, gender equity. Mothers noted the importance of finding a husband who would allow them to work outside the home and of working not in the informal sector as domestic servants, as so many of their generation and their mothers’ generation had, but in a shop or café, where they would, as one woman said, “accrue benefits and be treated well.” At times supported by progressive husbands and at other times prideful single mothers, many of these women had been politically active during the ‘70s and ‘80s when a range of grass-roots movement, including shantytown neighborhood organizations, gained force, and converged in the lead-up to ousting Brazil’s dictatorship.

Such ideological and experiential heterogeneity has generated more strain within families and communities than was the case just a generation ago. So-called “modern girls” often became the object of their neighbors’ gossip and were criticized for being snobby, even classist. Many lost childhood friends as a result. To mitigate against such difficulties, some ultimately rejected their shantytown origins, socializing only with schoolmates and waiting for the time when they might be able to move to another neighborhood. These women found comfort in endorsing the view that their personal successes had resulted from the opportunities of *adolescence*, which they spoke about as a kind of “new awakening” and social–psychological blank slate. By referring to more essentialized notions of their adolescences, they effectively justified the moral/personal suitability of their “modern” life choices and dampened the insecurities they felt about their future. Conversely, women who ultimately found nothing but disappointment with the conflict-ridden nature of their school experiences often left school pregnant, recoiling from social strife and entering into what they later came to argue was a more morally upstanding “traditional” life. Importantly, they typically asserted that the psychological notions of “adolescence” they had encountered in school were wholly inaccurate renditions of their experiences.

## Epistemic struggles

The social and moral stakes of adolescence clearly intensified in the 1980s and ‘90s. Reminiscent of Hacking’s “cultural polarity” vector, I suggest that parents and young people adopted “socially constructivist” and “essentializing” positions vis-à-vis “adolescence” as a way of wrestling with the merits and drawbacks of “modern life.” This “epistemic wrestling” became particularly meaningful for a subset of young women who were deeply unsatisfied with the prospect of either reproducing their “traditional” origins or becoming “modern.” Already as young teens, these women had been disapproving of the *machismo* their male friends showed, of girls they knew who had married their way “out of the shantytown,” turning their backs on their childhood friends, and of teachers and their classist ways. They wanted to stay in school, get better jobs, and enjoy gender equity, but not the classism that seemed so integral to such emancipation. They wanted time to date boys and not commit to marriage but they did not want the risk of singlehood and childlessness. Adolescence for these women acquired unique epistemic significance and productivity. It became neither a mere construct of elitism to be rejected nor an unproblematic pathway toward a better life, but a system of values to be reckoned with—a way of experimenting with and deconstructing the social norms to which it is tied. Seeking a space that defied the “traditional” and the “modern,” these women became quintessential *bricoleurs*.

To demonstrate this bricolage work, allow me to introduce Carolina, a young woman first sent to her school’s psychologist when she was 14.^5^ Her teachers described her as an “argumentative” and fitful adolescent struggling with low scholastic achievement. In contrast to many young people who respond to such suggestions by withdrawing from schooling, Carolina immersed herself more intensely in school life and began visiting the psychologist. But she was also not your typical “upwardly mobile” compliant student, for she approached therapy with a view to airing her complaints. She asked the psychologist, for example, why the teachers thought *she* needed help when other class mates with low grades never were referred. Why, she asked, did the school provided education sessions on “adolescent development” and “healthy behaviors” instead of important issues like violence, drugs, and jobs?

In her sixteenth year, in 1998, as many of her friends drifted away from school and began dating older men who had long left school, Carolina started dating a boy of her own age from school. Her choice reflected a conscious decision to avoid dating choices that teachers and parents often disavow, and she found him appealing because he was “modern” in his views and thought girls were entitled to do everything boys did—drink, party, and even work—if they wanted to. Ironically, Carolina’s romance, now plainly visible in school, became the object of growing concern. Her teachers feared she would be further diverted from school work and they suggested more intensive psychological support. Carolina’s friend told me, “They are worried about her because if you are not progressing in school, they say it can only mean that you are or will be making children at home.”

Carolina’s frustrations only grew: “As soon as I hooked up with Marcio,” she said, “they kept saying that I didn’t seem interested in my studies anymore….But all girls date!” Yet even at this crossroads, Carolina was not totalizing in her rejections and sought rather to intermix, even hybridize, the social-symbolic repertoire typically associated with adolescence. On the one hand, she argued that the “suggestions” made by teachers and school psychologists were classist and gendered. She told me that while “better off” kids are treated like fragile *adolescents* and given all sorts of freedoms, shantytown kids are treated like *aborrecentes* (a pun on the word “adolescence” which means “unlikable youth”) and curbed at every step. “What those [psychologists] say,” she continued, “is *cheio de frescura* (pretentiousness).” At the same time, she often said she “felt” like an adolescent and explained that her way of “growing up” was more extroverted than for many girls, more similar to that of boys. Adolescence helped legitimize her gender nonconformity. It became a “good to think with” object *because* she remained in school and used the adolescence-schooling nexus as an experience-near microcosm of a world she wanted to scrutinize.

A series of conflict-ridden practices often ensued on the heels of experiences such as Carolina’s. Repeatedly, I observed young women’s provocative bricolage practices looping into an intensifying psychologically reductionist framing of their life-worlds, especially in large “problem” schools situated near newly established shantytowns. In these schools, I found psychologists positing a theory for young women’s predicaments that was antithetical to all I had known about social psychiatry in Pelotas. Carolina’s behaviors, the school psychologist explained, indicated more than scholastic issues and the typical challenges associated with life in poverty. “It’s not just her poor school achievement and prolific dating that worry us,” Carolina’s teacher told me, “It’s also her agitation and attention problems, a tendency, even, toward aggression.” Carolina, I was told, was experiencing incongruence in the cognitive, social, and sexual dimensions of her development, and this “truncated” form of adolescence increased her risk for emotional agitation, mental morbidity, sexual risk taking, and teen pregnancy.

Consistently, I found that young women who defied social norms were more likely to elicit this kind of psychologized theorizing. Marisa, another case in point, had begun working with her mother from a young age at a café in the city center and her mother allowed her to keep some of the money she earned. Marisa relished in the independence this afforded her, for it meant, amongst other freedoms, that she did not need a boyfriend to pay for her entry to local night clubs. Ana and Joice, two other young women, were allowed to hang out on the streets in their neighborhood with the local boys, with whom they managed to maintain their status as peers rather than prospective dates. Though these young women’s mothers encouraged such unconventional forms of child-rearing, psychologists and teachers tended to link these young women's behaviors to psychological agitation and developmental incongruence.

The notion of sexual and cognitive developmental disjuncture that surrounded these cases is known as the “temporal gap theory” and dates back to early twentieth century Western European medicine and psychiatry (Koffman 2012). In Pelotas, a resurgence in interest in temporal gap theorists, most notably James Tanner (1955), first took place not among psychiatrists, but among pediatricians and obstetricians interested in studying the relationship between the population-based lowering of the age of menarche and the temporal widening of the interval from sexual to psychological maturation. In the early 1990s, several researchers interested in this question conducted studies that measured and correlated physiological, biological, and socio-psychological maturation levels in young people (Zerwes 2004). They found that developmental disjuncture was etiologically critical for a host of psychological and health outcomes. Over time, this biophysiodevelopmental perspective became increasingly popular among psychologists and educators. “I always tell young people,” one pedagogic advisor explained “that there is a big difference between being biologically prepared for reproduction and having the psychological maturity needed to become a parent. These kids don’t know if they are children or adolescents and this leads to developmental agitations, *indisciplina* (lack of discipline)… problems with school failure, aggression, sexual risk-taking, and drug and alcohol abuse.”

As temporal gap theories gained traction, the psychologization of adolescent sexuality acquired an additional “good to think with” layer, re-invigorating long-standing epistemic controversies to which many in the psychiatric community were committed. Critics of this theorizing, senior psychoanalytic psychiatrists among them, explained that “temporal gap” theories were akin to the more conservative “decontextualized one-person theories of development” associated with Freudian ego-psychology that, they argued, had long fallen out of favor among psychiatrists. Similarly, leading epidemiologists from the local Federal University’s Department of Social Medicine (DSM), a department renowned for its expertise in the “epidemiology of inequity” voiced concerns that the growing focus on adolescent development was no more than a strategic fad. As one prominent physician from the DSM told me,

Adolescence health?! Ah, yes, this has become fashionable. But it’s a question of how the physician constitutes his market. Adolescents don’t really get sick [or] die in great numbers, so really it becomes an issue of teen pregnancy, drug use, school problems… but are these psychological problems? Will we resolve them with adolescent medicine or adolescent psychiatry? I think not!

Researchers from the DSM began pushing for attention to the social forces shaping young people’s lives, including poverty and a substandard educational system, and they cautioned against making too much of “early intervention” programs centered on sexual development. In the late 1990s, they initiated an ethnographic study on teen sexuality nested within the 1982 longitudinal cohort study. Teen pregnancy, they discovered, did not always cause a sense of impending doom among young women. For many families, it was school failure and tense relationships with teachers that were fraught with turmoil. A survey conducted in 2001 with the cohort participants, then 19 years old, found that 25 percent of all teen pregnancies had been planned, 93 percent brought the mother “happiness,” and 60 percent occurred within the context of cohabitation/marriage or plans for future marriage. This research countered the well-rehearsed premise that teen pregnancy is a key *cause* of school abandonment and showed, rather, that scholastic difficulties tend to precede (and thus possibly contribute to) pregnancy (Gonçalves and Gigante 2006).

Epistemic struggles also intensified outside of the world of expertise, as young women began using the language of adolescence to negotiate gendered and classed power dynamics in their everyday relations. Long-standing quarrels between *certinha* (upstanding) and *froxa* (morally loose) girls became increasingly framed in the language of psychology. I once observed one such “good” student say as her rival walked by, “That one there, she has become *froxa* with the boys and is failing in school. Her *cabeça* (mind) isn’t on straight—you’ll see, she’ll be pregnant in six months. The pregnant ones say they actually want to, but I think it’s because they *falta cabeça* (lack a good mind, maturity).” In contrast, young women who left school to “marry” and set up their own family actively denigrated young women like Carolina for their gender nonconformity and “demonstrative” dating, claiming such behaviors to be not just improper but also psychologically harmful. Such comments, not at all unusual, indicate the extent to which the intentionality and moral status of teen pregnancy became contested.

Significantly, several young women turned to more critical elements in the psychiatric community for support in legitimizing their epistemic positions and life-choices. Both Marisa and Ana, for example, told me that they discussed their struggles with classism and gender restrictions with a psychiatrist they sought of their own accord. Joice’s mother, who had long been seeing a social psychiatrist, was a key support for her daughter’s emerging critical urgings. Similarly, when Carolina’s teachers and peers began to hone in on her “prolific dating,” one of her closest friends, Andrezza, who had been visiting a social psychiatrist, told her in no uncertain terms: “Don’t believe there is anything wrong with you. It’s this world … full of *preconceito* (prejudice).” Carolina, who heeded carefully her friend’s views, later told me, “Listen, yes, my nerves attack me, but no more than anyone else. It’s this life. I am very *consciênte* (conscious and conscientious) of what I do.” Supported by Andrezza, Carolina began to confront social and gender norms more explicitly. In time, she became subtly satisfied with the diagnostic language of “behavioral impulsivities” that was beginning to form around her partially because this language, typically associated with boys’ problems, acquired the ability to shift social and moral givens.

Whether reinforced or questioned—or rather, *because* both reinforced and questioned—a psychological language of adolescent development and sexuality gained traction. By early 2000s, informal pockets of proto-scientific activity began cropping up. The psychologist in Carolina’s school, for example, began documenting the relationship between agitation, premature sexual maturation, and school achievement in students whom she asserted were likely to become teen mothers. “Sex amongst disadvantaged teens,” she told me, “has become increasingly demonstrative… a way of shocking adults and saying I am here, I exist, I am defiant.” In another school, a psychopedagogic advisor responsible for documenting referrals to the psychologist explained, “If these kids want to argue with you, they will.… Any differences with their peers, they become agitated, immediately turn to aggression. It shows in their sexual urgings.” The head teacher in another school told me that the data her pedagogic specialist was collecting suggested that a pregnant teen in the classroom “deregulates disciplinary control” among nonpregnant peers. Inspired by such “data,” I began to notice an inversion of conventional causal theories on the social determinants of mental health, with school staff positing that socioeconomic forces do not determine mental life but rather simply exacerbate preexisting biopsychological tendencies.

Latour (1996) reminds us that some scientific projects come into being not in canonical ways but through the accumulation of “little resistances” and “solidities”—small *co-constituting* steps, bubbling up from the everyday. Science *gives itself* a context. The more young women were exposed to the notion that their sexualities and psyches might be underpinned not by romance or budding adulthood but by developmental impulsivity, the more their desire to affirm their normality gained momentum, and the more they turned toward specific behaviors—acting out, “prolific” dating—to assert their right to be normal in these ways. Adolescent pregnancy itself, I will show below, occurred as one in a long line of such behavioral assertions. Young women’s “little resistances” reaffirmed the etiological centrality of the adolescent “agitated” and “aggressive” profile that had begun to crystallize, which fomented, in turn, the need for more psychological intervention. And so the looping gained depth and breadth, providing a “self-vindicating” justification for the project’s emerging scientific form (Hacking 1995).

## Ontological politics

The epistemic struggles in which young women engaged were not mere conceptual practices turning on the internalization—and rejection—of psychological language. These women did not just embody the weight of the social and institutional worlds that surrounded them, with end results that merely reinforced existing power structures. For this reason, I turn here to questions of ontology. Where “embodiment” theory has tended to focus on how social life gets “under the skin,” ontology more readily captures its projection back out. A psychologized language of adolescence became “good to think with” precisely because it provided a conduit for experimenting with new ways of being and becoming in the world (Biehl and Locke 2010). To toy with becoming or not becoming “an adolescent,” which at that time was so imbued with epistemic diversity and tension, engendered a “politics of ontology,” to use Holdbraad and colleagues’ term (2014), through which young women like Carolina sought to “alter from themselves.” These women were able to alter ontologically “from themselves”—and from the normative trends that might otherwise be their destinies—not *in spite* of their encounters with psychologized languages, but in part *because of* these encounters. As Foucault tell us, some forms of critique engage not “question(s) of the analytic of truth but…. [of] what could be called an ontology of the present, of present reality, an ontology of modernity… [and] of ourselves” (2010:95).

Epistemic openings, tensions, and bricolage work meld into ontological opportunities. Though a form of release, such bricolage work was difficult and strained, and in this sense, I use the term ontology to refer also to the ways in which emotional strife unfolded in these women’s lives. Recall that only *some* young mothers became persistently distressed. Others did not. Many forces account for this difference, the most consistently researched being single motherhood, and the downward economic mobility that families already living in poverty often experience when becoming parents. Yet, as I have shown elsewhere (Béhague et al. 2012), there is a great deal more to this picture. Teens who transitioned into their role as mother with minimal emotional struggle were relegated and relegated themselves to the world of “tradition.” They justified the moral suitability of their life choices by moving symbolically *and* physically away from institutional environments and relationships—for example, school, clinic, and places of employment—that framed their ways of being in the language of psychological impulsivity. When they did struggle, they articulated their difficulties in reference to the challenges of motherhood and family dynamics, not nerves and depression.

In contrast, for women who sought a social and moral grey zone, who wanted to remain institutionally visible and vocal, emotional suffering became integral to the kind of motherhood they eventually embraced. Vital to their broader political struggles, their nerve-provoking pregnancies unfolded as semi-accidental and semi-intentional occurrences that acquired layer upon layer of emotive bodily experiences and meaning, crystallizing into a new biosocial form (Lock 2015). Pregnancy became so ontologically saturated as to become one of the “thickest” of happenings I have come to know (Geertz 1973). A particularly absorbent and politicized “kind” of teen motherhood, it became a “moving target” (Hacking 1999), elusive to scientific classification but also always incrementally absorbing *and feeding* the epistemic tensions that are so constitutive of psychiatric expertise in Pelotas.

To demonstrate how these unexpected turns of events unfolded, let me turn to young women’s early experiences with menstruation, contraception, and sex. Menstruation in Brazil is a finely tuned signifier of reproduction and fertility. Women speak of “possible pregnancies” and “possible early miscarriages” as “menstrual delays” and “menstrual cleansing” respectively, even when they have not confirmed a pregnancy (Sanabria 2016).^6^ For some women, to experience a menstrual delay and be “possibly-pregnant” became a way figuring out what “kind of life” they truly wanted—modern, traditional, something in-between? As Marisa’s older sister explained, aspiring “moderns” typically respond to menstrual delays fearfully, by recommencing the contraceptive pill and intensifying their studies, while “traditional girls”—especially those who felt marginalized in school—responded by testing their boyfriends’ commitment to possible marriage. Carolina, Marisa, and Ana, among others, went so far as to experiment with stopping contraceptive use for a few days, and though they justified this decision by arguing that the body must be cleansed of the pill’s toxicity, underlying these experimental moments was a clear sense of uncertainty about the merits and perils of “modern” life. It was precisely when Carolina became overwhelmed with doubt about her life trajectory—“Will schooling bring me a better life?” she asked, “Do I really have psychological problems?”—that she stopped the pill and began with the *tabelinha* (rhythm) method, charting her menstrual cycle and avoiding sex on her fertile days. Her boyfriend, Paulo, accepted her decision and their emotional commitment to the relationship grew, as did their public demonstrations of this commitment. When Carolina experienced her first menstrual delay, she toyed with the idea of giving up all scholastic, social, and political ambitions– yet she never did.

The protagonists described in this article reacted to their intimate relationships and menstrual delays by seeking to collapse the traditional-modern bifurcation altogether. Almost all remained in school and persisted with dating, at times falling in love, despite the fact that they became the object of growing “risk infused” gossip in school. Some of Carolina’s classmates, for example, noting her academic difficulties and relationship with Paulo, predicted she would soon become pregnant. Her teacher swooped in to provide more psychological support, but Carolina’s public contestations only intensified. One afternoon, as I sat with Carolina and her friends listening to the usual banter about boyfriends and sex, Carolina provocatively announced she was using the *tabelinha* method. When one of her friends balked, she quickly countered and affirmed that the *tablinha* method was as safe as the pill, and added, “Pregnancy is not the end of the world, you know.” She held firm, saying she was not being impulsive and that she would still be “free” to pursue what she wanted in life even were she to become a young mother. “Besides,” she continued, “Teachers say we will end up on the streets if we leave school, but that’s not true. I know plenty of people who have good jobs who didn’t finish school; and one who got into college and can’t find a job!”

When Carolina discovered she was pregnant a few months into her eighteenth year (in 2000), she felt intense fear of the future and intractable commitment to motherhood. In one sense, her struggles to succeed in school and to actually have an “adolescence” had materialized into a pregnancy that underscored her failure to do so. Carolina did not, however, accept this interpretation of her potential motherhood. Instead, her pregnancy became a highly meaningful moment of politically infused ontological bricolage. Whereas most young women who become pregnant either leave school or get a clandestine abortion, Carolina remained committed to her maternity, education, *and* future employment. Such ontological hybridity was consistently the case for all these young women. Some did their best to keep up in school despite increased absences; others transferred to night school and a few returned several months after the birth of their child.

Similarly, all made unexpected and meaningful decisions about where and with whom to live and socialize. Carolina and Paulo did not build a shack in the rear of one of their parents’ plots, accepting a position of dependency as many young couples do. Instead, they began to stockpile small amounts of wood for their future one-room family home to be built on the border of the shantytown. Ana and Beto, her future husband, were supported by his upwardly mobile parents to move away from the shantytown and take up residence in a downtown apartment. Ana was uncomfortable with this decision, so she made a point of remaining in her original school situated close to her natal shantytown and routinely visited her old childhood friends. Marisa, who did not remain in a relationship with her boyfriend, continued to live with her mother in their one-room home while working part-time and attending school. She told me several times that her daughter was her life’s inspiration, and this meant thinking first about gaining her own independence. If her mother was able to avoid becoming a house maid, she would take this further and one day become a teacher.

**Figure 1.**
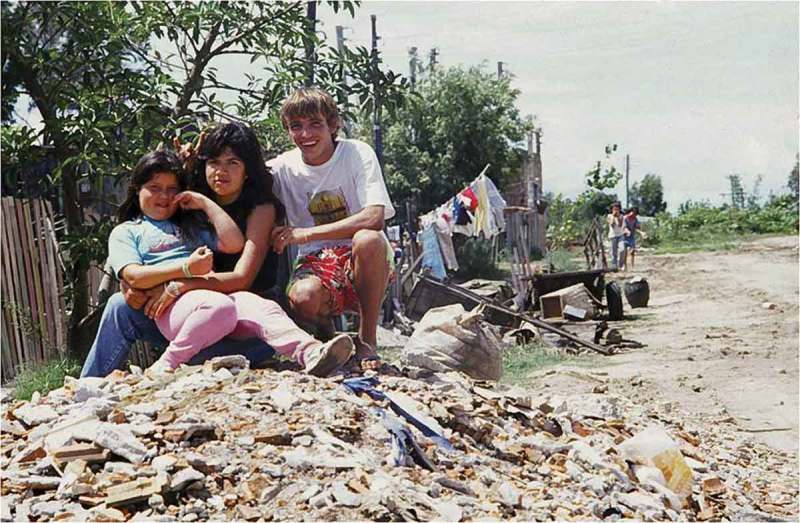
Carolina with her cousin and boyfriend in front of Carolina’s home.

These women’s form of politics was less insurgent than “tinkering,” as they sought to turn the most basic of social expectations and moral positions inside out. In an article on the problematization of teen pregnancy in public health, Hacking has argued that critical contestations such as these, which he termed a “reworking” of classificatory science and its causal assumptions, are suffused with a desire for “pride and self-control” (1999:80). The most poignant of these pride seeking moments struck me when, while on a stroll on her street in the shanty with Carolina, Paulo, and her young cousin, I asked Carolina if I could take a picture of her (see Figure 1). Her response differed significantly from that of most young women, who typically requested that I come back another day so they could tidy their homes and put on their best clothes. Instead, Carolina said, “come on, let’s get on top of this rubble for the picture. I have nothing to hide.” At the time of the picture, she was a few months pregnant, though she had disclosed this to no one other than Paulo, her eldest sister, and me. Carolina was well versed in the reasons for my presence in her life. Certainly, she imagined an entrenched upper-middle class readership at the end of my pen. With this readership in her mind, she used my camera as a conduit for her message, intrepidly exposing her personal politics to an unsupportive world from which so many others recoil.

Such reworking intensified during pregnancy and into motherhood to include, most notably, a reclassification of pregnancy as having been planned and desired. Marisa, Ana, Carolina and several others told me repeatedly that they did not feel like a “child having a child.” On the contrary, motherhood gave them the courage and sense of purpose to “conquer” their life, complete their education, and confront the social injustice with greater resolve. When nearing the end of her pregnancy, Carolina recounted her life story, saying “I don’t regret becoming a mother…it has been the best experience. It has made me mature. Sure, my pregnancy was ‘unplanned’ but really, it was a conscious thing.” Noting that she and Paulo “knew” she had not been taking the pill regularly, she added, “information about contraception is everywhere. Those who get pregnant, it’s because they want to, not because of ignorance. *Quem ta na chuva é pra se molhar* (You can’t walk in the rain without getting wet).”

Carolina’s pregnancy was technically “accidental,” yet she was one of the 25 percent of women who defiantly stated in the 2001 epidemiological survey of the 1982 birth cohort that her pregnancy had been actively planned. While fulfilling and “not-accidental,” motherhood became a strained experience for these women, a constant reminder of their relentless struggle to *be otherwise*. Several expanded the scope of their reworking, adopting positions of leadership in their communities, spending time on the streets, engaging in public debates at the local corner shop, and supporting girlfriends seeking advice on a range of issues, from overbearing boyfriends to the alienating expert-infused world of education. And yet, there was no pat middle-class or working-class community in which these women could find a sense of belonging. They often felt alone, even estranged. “I can’t talk to anyone like I can talk to you,” Carolina told me, “My friends look to me for confirmation that we are right, that we have the same *cabeça* (way of thinking), but….”

Carolina began suffering from *nerves* problems and at times frank *nerve attacks*, characterized by bodily tremors, crying fits, and an inability to sleep or eat. The same was true for Marisa, Ana, and several other young mothers. Remaining in school often compounded their *nerves*, especially night schools which tend to be of poor quality and infamous for having high levels of violence and illicit drug use. Nerves and continued psychological intervention in schools tacked back and forth, entrenching both morbidity and diagnosis. This was in the mid-2000s, and right around the time a more categorical diagnostic language was beginning to take shape in Pelotas. As these women’s understanding of their own woes shifted from that of *nerves* to “depression” and “anxiety,” they began to wonder if their own unresolved adolescences might be at the root of their emotional turbulence. While several took temporary refuge in the explanatory simplicity that a biomedical framing enables—divorcing their “depression” from their life stories—they persevered in their determination to remake their lives and worlds. Within a few years, all ended up with a job of their own choosing, a higher level of education than many of their childhood counterparts, a strong sense of emotional anguish, and a contested psychiatric language with which to continue reworking their social–moral worlds.

## Budding bioscience

The epistemic beginnings of the science that crystallized during Carolina’s early adult years lay not in the straightforward importation of research from the global sphere, nor from the work of local professional “masterminds.” It wasn’t until the mid to late 2000s that diagnostic and screening tools developed largely by Anglophone researchers were validated for the Brazilian context and incorporated into Pelotense research. It was only then that publications began using discrete diagnostic measures taken from biomedical psychiatry, making explicit reference to neuropsychiatric theories of cause and documenting statistically significant associations between teen pregnancy and comorbidities such as drug/alcohol abuse and conduct disorder (Chalem et al. 2012; da Silva et al. 2007; Pinheiro et al. 2007). Only by the latter part of the 2000s did the category “post-partum depression” become more common place (Moraes et al. 2006).

Rather, the beginnings of this science materialized from the ground up once a critical mass of “little solidities, little durabilities, little resistances” (Latour 1996:45) had been reached and only as the first inklings of a localized protoscience became ontologically looped into a series of social, pedagogic, political, economic, moral, and clinical concerns and problems, not least of which was the adolescence-education nexus. This ontological looping is on some level striking. Carolina had intended to become anything but pregnant as a teenager, and she certainly did not foresee that her eventual young motherhood would become imbued with anxiety-provoking political commentary. Yet as Hacking (1995) notes, knowledge of the “kind” and the “kind” emerge hand in hand, each egging the other on: as fields of description change, so do the possibilities for personhood and intentional action. For these women, such possibilities erupted from an array of “durabilities and resistances”—small and not-so-small moments that took shape well before their sexual debut: repeated referrals for psychological care; encounters with the emergent language of adolescent development and impulsivity; classist denigrations and unpalatable gender norms; these and other such moments materialized iteratively along with romance, sex, classism, schooling, motherhood, nerves, and depression—all part of an incrementally emergent ontological politics saturated with “biosocial” entanglements (Raikhel 2015).

Current academic debates on the relationship between teen pregnancy and psychiatric morbidity are caught in a polarizing “essentialist” versus “social constructivist” gridlock. As psychiatric-epidemiological claims that adolescent pregnancy is determined by and leads to neurodevelopmental morbidity become entrenched, so do sociological analyses claiming this knowledge base to be politically motivated “social constructionism,” with studies showing that preexisting poverty and not age of pregnancy account for subsequent mental illness (Lawlor and Shaw 2007). In this article, I have endeavored to investigate this polarization as an object of analysis rather than to rely on it heuristically. To use Karen Barad’s phrasing (2003), I have shown how “causal relations”—between scientific constructs and debates, social inequity, personal politics, adolescence, and mental distress—intra-actively produced new ontologies which in turn fed the institutionalization of psychologized approaches to adolescent sexuality.

In this sense, the way in which reductionist theories of adolescent development became interwoven with young women’s sense of injustice, generally, but also as it related to use of these theories, posed a significant threat to the ideals of social psychiatry. At the same time, the co-constitution of science and person-kinds materialized not *in spite of* the politicizing work of critical psychiatrists, psychologists, and social epidemiologists, but partially *because of* it. Constructivist and essentializing logics were actors in the field of action. The critical reflexivity that permeated throughout, the contested status of psychiatric knowledge about adolescence, and the fact that the transition from childhood to adulthood has undergone rapid structural change have rendered adolescence uniquely “good to think with.” Thus, young women’s agonizing *reworkings* did more than reproduce the conditions of their oppression. Rather, their *bricolage* efforts embraced a form of becoming, an ontological politics, that mobilized—to recall Foucault’s answer to the question, “What is critique?”—“the art of not wanting to be governed quite so much or quite like that” (Foucault and Lotringer 1997:46).

## Notes

1. I selected this group of young people randomly from an ongoing epidemiological birth cohort study initiated in 1982 by colleagues at the Federal University of Pelotas (Victora et al. 2011). Fieldwork, comprised of participant observation and repeated semi-structured and informal interviewing with youth and their families, was conducted by myself, another anthropologist, and four research assistants. Random sampling was used not to conduct probabilistic analyses, but to capture an array of life experiences, including those of particularly isolated and introverted youth.

2. Since 2007, I have kept in touch with a handful of these young women, following their lives into adulthood.

3. Of the 25 young mothers from the cohort, 13 entered motherhood with minimal emotional upheaval and 12 experienced considerable distress, which in their adult years they came to refer to as nerves, anxiety and depression.

4. While few parents openly sanction premarital sex, it is broadly acceptable for young girls to become sexually active by around age 16, so long as *single motherhood* – the more problematic issue at hand – is avoided. Young women have more reproductive agency than is often assumed by public health researchers; contraceptive-utilization rates tend to be high, nearing of 80–90 percent of sexually active teens (da Rocha et al. 2007).

5. In this article, I focus primarily on the story a single young woman Carolina to underscore the longitudinal dynamics at play, though her experiences were remarkably similar to the 12 young mothers who experienced prolonged emotional distress. All names used are pseudonyms.

6. Although abortion is illegal, many women use of the relatively safe abortive medication misoprostol in the first months of pregnancy.

## References

[CIT0001] Ariès P. 1962 Centuries of Childhood: A Social History of Family Life. New York: Vintage Books.

[CIT0002] Barad K. 2003 Posthumanist performativity: Toward an understanding of how matter comes to matter. Signs 28(3):801–831.

[CIT0003] Béhague D. P. 2009 Psychiatry and politics in Pelotas, Brazil: The equivocal uses of “conduct disorder” and related diagnoses. Medical Anthropology Quarterly 23(4):455–482. 20092054 10.1111/j.1548-1387.2009.01073.x PMC2810432

[CIT0004] Béhague D. P., H. D. Gonçalves, D. Gigante, and B. R. Kirkwood 2012 Taming troubled teens: The social production of mental morbidity amongst young mothers in Pelotas, Brazil. Social Science & Medicine 74(3):434–443. 22196249 10.1016/j.socscimed.2011.10.014 PMC3272444

[CIT0005] Biehl J. and Locke P. 2010 Deleuze and the anthropology of becoming. Current Anthropology 51(3):317–351.

[CIT0006] Bowler P. J. 1989 The Invention of Progress: The Victorians and the Past. Oxford, UK: Basil Blackwell.

[CIT0007] Carrara S. L. and Russo J. A. 2000 A psicanalise e a sexologia no Rio de Janeiro de entreguerras: Entre a ciencia e a auto-ajuda. Historia, Ciencias e Saude- Manguinhos 9(2):273–290. 10.1590/s0104-59702002000200003 12418491

[CIT0008] Chalem E., S. S. Mitsuhiro, P. Manzolli, M. C. Barros, R. Guinsburg, N. Sass, R. Laranjeira, et al 2012 Underdetection of psychiatric disorders during prenatal care: A survey of adolescents in Sao Paulo, Brazil. Journal of Adolescent Health 50(1):93–96. 22188840 10.1016/j.jadohealth.2011.03.012

[CIT0009] Comaroff J. and Comaroff J. 2006 Reflections on youth, from the past to the postcolony *In* Frontiers of Capital: Ethnographic Reflections on the New Economy. Fisher M. and Downey G., eds. Durham, NC: Duke University Press.

[CIT0010] da Rocha C. L. A., B. L. Horta, R. T. Pinheiro, A. L. S. Cruzeiro, and S. Cruz 2007 Use of contraceptive methods by sexually active teenagers in Pelotas, Rio Grande do Sul State, Brazil. Cadernos de Saúde Pública 23(12):2862–2868. 18157328 10.1590/s0102-311x2007001200007

[CIT0011] da Silva R. A., B. L. Horta, L. M. Pontes, A. D. Faria, L. D. D. M. Souza, A. L. S. Cruzeiro, and R. T. Pinheiro 2007 Psychological well-being and adolescence: Associated factors. Cadernos de Saúde Pública 23(5):1113–1118. 17486234 10.1590/s0102-311x2007000500013

[CIT0012] Foucault M. 2010 The Government of Self and Others (Lectures at the College de France 1982–1983). New York: Palgrave.

[CIT0013] Foucault M. and Lotringer S. 1997 The Politics of Truth. Los Angeles, CA: Semiotext(e).

[CIT0014] Geertz C. 1973 Interpretation of Cultures. New York: Basic Books.

[CIT0015] Gonçalves H. D. and Gigante D. P. 2006 Work, schooling, and reproductive health: An ethno-epidemiological study of adolescent women belonging to a birth cohort. Cadernos de Saúde Pública 22(7):1459–1469. 16791345 10.1590/s0102-311x2006000700010

[CIT0016] Hacking I. 1995 The looping effect of human kinds. *In* Causal Cognition D. Premack Sperber, D., and Premack A., eds. Pp. 351–383. Oxford, UK: Oxford University Press.

[CIT0017] Hacking I. 1998 Mad Travelers: Reflections on the Reality of Transient Mental Illness. Charlottesville, VA: University Press of Virginia.

[CIT0018] Hacking I. 1999 Teen pregnancy: Social construction? *In* Teen Pregnancy and Parenting: Social and Ethical Issues. Wong J. and Checkland D., eds. Pp. 71–81. Toronto, Canada: University of Toronto Press.

[CIT0019] Hall G. S. 1905 Psychology of Adolescence. Englewood Cliffs, NJ: Prentice Hall.

[CIT0020] Harding S. 1996 Science is ‘good to think with.’ Social Text 46/47:15–26.

[CIT0021] Heilborn M. L., Brandao E., and Cabral C. 2007 Teenage pregnancy and moral panic in Brazil. Culture, Health & Sexuality 9(4):403–414. 10.1080/13691050701369441 17612959

[CIT0022] Holbraad M., Pedersen M. A., and de Castro E. Viveiros 2014 The politics of ontology: Anthropological positions *In* Fieldsights - Theorizing the Contemporary, Vol. 2015 Cultural Anthropology Accessed from: https://culanth.org/fieldsights/462-the-politics-of-ontology-anthropological-positions

[CIT0023] Jasanoff S. 2012 Genealogies of STS. Social Studies of Science 42(3):435–441.

[CIT0024] Koffman O. 2012 Children having children? Religion, psychology and the birth of the teenage pregnancy problem. History of the Human Sciences 25:119–134. 27656726 10.1177/0952695111426383

[CIT0025] Latour B. 1996 Aramis, or the Love of Technology. Cambridge, MA: Harvard University Press.

[CIT0026] Lawlor D. A. and Shaw M. 2007 Is teenage pregnancy a public health problem. *In* Pregnancy Teenage and Reproductive Health P. Guthrie Baker, K., Hutchingson C., Kane R., and Wellings K., eds. Pp. 43–58. Dorchester, UK: Royal College of Obstetricians and Gynaecologists.

[CIT0027] Lesko N. 2001 Act Your Age!: A Cultural Construction of Adolescence. London: Psychology Press.

[CIT0028] Lévi-Strauss C. 1966 The Savage Mind. Chicago, IL: University of Chicago Press.

[CIT0029] Lock M. 2015 Comprehending the body in the era of the epigenome. Current Anthropology 56(2):151–177.

[CIT0030] Luker K. 1996 Dubious Conceptions: The Politics of Teen Pregnancy. Cambridge, MA: Harvard University Press.

[CIT0031] Moraes I. G. S., R. T. Pinheirob, R. A. da Silva, B. L. Hortac, P. L. R. Sousab, and A. D. Fariab 2006 Prevalência da depressão pós-parto e fatores associados. Revista de Saúde Pública 40(1):65–70. 16410984 10.1590/s0034-89102006000100011

[CIT0032] Neubauer J. 1992 The Fin-de-siecle Culture of Adolescence. New Haven, CT: Yale University Press.

[CIT0033] Pinheiro K. A., B. L. Horta, R. T. Pinheiro, L. L. Horta, N. G. Terres, and R. A. da Silva 2007 Common mental disorders in adolescents: A population based cross-sectional study. Revista Brasileira de Psiquatria 29(3):241–245. 10.1590/s1516-44462006005000040 17713704

[CIT0034] Povinelli E. A. 2011 The governance of the prior. Interventions: International Journal of Postcolonial Studies 13(1):13–30.

[CIT0035] Raikhel E. 2015 From the brain disease model to ecologies of addiction *In* Re-Visioning Psychiatry: Phenomenology Cultural, Neuroscience Critical, and Global Mental Health L. Kirmayer J., ed. Cambridge, UK: Cambridge University Press.

[CIT0036] Rajan K. S. and S. Leonelli 2013 Introduction: Biomedical trans-actions, postgenomics, and knowledge/value. Public Culture 25(371):463–475.

[CIT0037] Rose N. 2013 The human sciences in a biological age. Theory, Culture & Society 31(1):3–34.

[CIT0038] Sanabria E. 2016 Plastic Bodies: Sex Hormones and Menstrual Suppression in Brazil. Durham, NC: Duke University Press.

[CIT0039] Schmidt R. M., C. M. Wiemann, V. I. Rickert, and E. B. Smith 2006 Moderate to severe depressive symptoms among adolescent mothers followed four years postpartum. Journal of Adolescent Health 38(6):712–718. 16730600 10.1016/j.jadohealth.2005.05.023

[CIT0040] Stepan N. L. 1991 “The Hour of Eugenics”: Race, Gender and Nation in Latin America. Ithaca, NY: Cornell University Press.

[CIT0041] Tanner J. M. 1955 Growth at Adolescence. Oxford, UK: Blackwell Scientific Publications.

[CIT0042] Tenorio F. 2002 A reforma psiquiátrica brasileira, da década de 1980 aos dias atuais: História e conceito. História, Ciências, Saúde – Manguinhos 9(1):25–59. 10.1590/s0104-59702002000100003 12382632

[CIT0043] Victora C. G., E. M. Aquino, M. do Carmo Leal, C. A. Monteiro, F. C. Barros, and C. L. Szwarcwald 2011 Maternal and child health in Brazil: Progress and challenges. The Lancet 377(9780):1863–1876. 10.1016/S0140-6736(11)60138-4 21561656

[CIT0044] Zerwes E. P. 2004 Feminina Puberdade Revista de Medédina da Universidade Católica de Pelotas, Pelotas 2(1):43–47.

